# Epigenetic regulation of aging: implications for interventions of aging and diseases

**DOI:** 10.1038/s41392-022-01211-8

**Published:** 2022-11-07

**Authors:** Kang Wang, Huicong Liu, Qinchao Hu, Lingna Wang, Jiaqing Liu, Zikai Zheng, Weiqi Zhang, Jie Ren, Fangfang Zhu, Guang-Hui Liu

**Affiliations:** 1 State Key Laboratory of Membrane Biology, Institute of Zoology, Chinese Academy of Sciences, 100101 Beijing, China; 2 State Key Laboratory of Stem Cell and Reproductive Biology, Institute of Zoology, Chinese Academy of Sciences, 100101 Beijing, China; 3 University of Chinese Academy of Sciences, 100049 Beijing, China; 4 School of Biomedical Engineering, Shanghai Jiao Tong University, 200030 Shanghai, China; 5 CAS Key Laboratory of Genomic and Precision Medicine, Beijing Institute of Genomics, Chinese Academy of Sciences and China National Center for Bioinformation, 100101 Beijing, China; 6 Hospital of Stomatology, Sun Yat-sen University, 510060 Guangzhou, China; 7 Guangdong Provincial Key Laboratory of Stomatology, Guanghua School of Stomatology, Sun Yat-sen University, 510060 Guangzhou, China; 8 Institute for Stem Cell and Regeneration, Chinese Academy of Sciences, 100101 Beijing, China; 9 Advanced Innovation Center for Human Brain Protection, National Clinical Research Center for Geriatric Disorders, Xuanwu Hospital, Capital Medical University, 100053 Beijing, China; 10 Beijing Institute for Stem Cell and Regenerative Medicine, 100101 Beijing, China

**Keywords:** Diseases, Senescence

## Abstract

Aging is accompanied by the decline of organismal functions and a series of prominent hallmarks, including genetic and epigenetic alterations. These aging-associated epigenetic changes include DNA methylation, histone modification, chromatin remodeling, non-coding RNA (ncRNA) regulation, and RNA modification, all of which participate in the regulation of the aging process, and hence contribute to aging-related diseases. Therefore, understanding the epigenetic mechanisms in aging will provide new avenues to develop strategies to delay aging. Indeed, aging interventions based on manipulating epigenetic mechanisms have led to the alleviation of aging or the extension of the lifespan in animal models. Small molecule-based therapies and reprogramming strategies that enable epigenetic rejuvenation have been developed for ameliorating or reversing aging-related conditions. In addition, adopting health-promoting activities, such as caloric restriction, exercise, and calibrating circadian rhythm, has been demonstrated to delay aging. Furthermore, various clinical trials for aging intervention are ongoing, providing more evidence of the safety and efficacy of these therapies. Here, we review recent work on the epigenetic regulation of aging and outline the advances in intervention strategies for aging and age-associated diseases. A better understanding of the critical roles of epigenetics in the aging process will lead to more clinical advances in the prevention of human aging and therapy of aging-related diseases.

## Introduction

Aging is a slow but gradual process that is characterized by a continuous decline in the normal physiological functions of living organisms over their lifespan. With age, the body’s resilience decreases, making it more sensitive to aging-related diseases, such as neurodegenerative diseases and cancer, and increasing the risk of death.^1–3^ Although most organisms have a similar death curve and a higher mortality rate during aging, dramatic variance in aging rates can be observed within the same species. Taking honey bees as an example, although the queen bee and the worker bees are genetically identical, the queen lives on average ten times longer.^4^ Intriguingly, there are so-called “non-aging” organisms that have exceptionally long lifespans, exhibit no or late-onset aging-related declines in physiological abilities and are resistant to aging-related diseases, such as hydra and naked mole rats.^5–7^ These phenomena indicate that aging is a complicated process that may be regulated by a variety of different factors.

Numerous studies have revealed how aging occurs and how it is regulated by complex cellular and molecular mechanisms at different stages of life. Many factors affecting the aging process and longevity have been reported,^8–10^ including telomere shortening, nutrient sensing, mitochondrial dysfunction and oxidative stress, deterioration of DNA repair and accumulation of DNA damage, changes in protein homeostasis leading to the accumulation and aggregation of misfolded proteins, and changes in epigenetic regulation. The word “epigenetics” is derived from the Greek word “epi” and means “over” or “above” the genome. Epigenetics represents a reversible mechanism in regulating the function of the genome without altering the underlying DNA sequence of the genome; thus, the epigenome links genotype to phenotype, which plays an important role in modulating the aging process in response to environmental stimulation.

Epigenetic modifications are often reversible with the aid of epigenetic regulators, which lay the theoretical basis for aging modulation and make them promising targets for aging-intervention strategies. However, it was not until recently that a series of important studies have been carried out on epigenetic regulation and interventions for aging. In 1967, whole-genome DNA methylation was found to be related to the age of spawning salmon.^11^ Subsequent studies revealed that DNA methylation was generally downregulated in a variety of mouse tissues and human fibroblasts during aging.^12,13^ In 1987, the nucleosome occupancy in human skin fibroblasts was shown to decrease during aging, suggesting that chromatin configuration may change in the aging process.^14^ In 2010, histone methylation was first linked with life extension, and it was demonstrated that H3K4me3 demethylation in the germline boosted the lifespan of *C. elegans.*^15^ With increasing epigenetic evidence related to aging, in 2013, the concept of the “epigenetic clock” was proposed to link DNA methylation with biological age.^16^ In addition, RNA modifications and ncRNA regulation have recently been demonstrated to be involved in the regulation of aging.^17^ Furthermore, emerging single-cell chromatin modification profiling may provide molecular information with an unprecedented resolution of the relationship between epigenetics and aging in the future.^18–20^

**Fig. 1:**
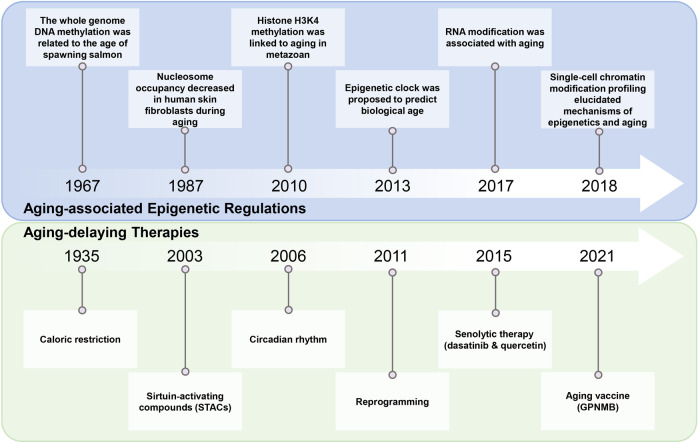
The history of studies on aging-associated epigenetic regulation and interventions

Understanding how aging is regulated by epigenetic factors greatly facilitates the development of aging-delaying therapies. Ever since the first report of caloric restriction (CR) to slow down aging in 1935, researchers have been exploring potential approaches to delay aging.^21^ One of these aging-intervention studies shows that the aging process can be delayed, and the healthy lifespan or healthspan can be extended by CR and lowering the basal metabolic rate.^22^ Another exemplary study is the discovery of resveratrol, an agonist of the longevity factor SIR2 of the sirtuin family, and its function in extending the lifespan of yeast.^23,24^ In addition, heterochronic parabiosis (HP) and circadian rhythm models have been found to be effective in identifying factors that delay aging.^25,26^ In 2011, senescent cells from centenarians or Hutchinson–Gilford progeria syndrome (HGPS) patients can be fully reprogrammed to a pluripotent state with a rejuvenated epigenome, suggesting the potential of reprogramming in the reversal of aging.^27,28^ In 2015, a combination of dasatinib and quercetin was identified to kill senescent cells selectively; hence they were named senolytic drugs.^29^ More recently, the concept of aging vaccines was proposed, and glycoprotein nonmetastatic melanoma protein (GPNMB) vaccination has been shown to decrease tissue senescence and alleviate aging-related phenotypes.^30^ However, how epigenetic mechanisms are involved in these aging-intervention approaches has just begun to be revealed (Fig. 1).^31^

In this review, we will discuss how epigenetic remodeling, including DNA methylation, histone modification, chromatin remodeling, RNA modification, and non-coding RNA regulation, is regulated during aging. We will also introduce current therapeutic strategies to delay aging, including small molecules, reprogramming, active health, and many other epigenetic-associated approaches.

## Epigenetic regulation of aging

**Fig. 2:**
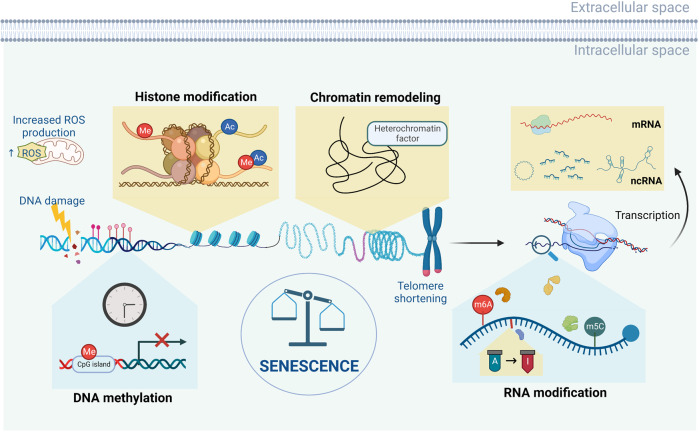
An overview of the aging epigenome. During aging and the emergence of cellular senescence, a series of epigenetic changes occur in cells, including alterations in DNA methylation, chromatin remodeling, histone modification, RNA modification, and ncRNA regulation

Accumulating evidence from invertebrate and vertebrate organisms, tissues, and in vitro systems links aging with epigenetic mechanisms. In mammals, there are global and local DNA methylation changes in the genome during aging. Additionally, there is a general loss of histones as well as global chromatin remodeling in all aging models. RNA modification and ncRNA regulation also play essential roles in cellular senescence via post-transcriptional regulations. Studies on how these epigenetic mechanisms regulate individual aging can provide targets to delay aging and rejuvenate aging organisms (Fig. 2).

### DNA methylation

**Fig. 3:**
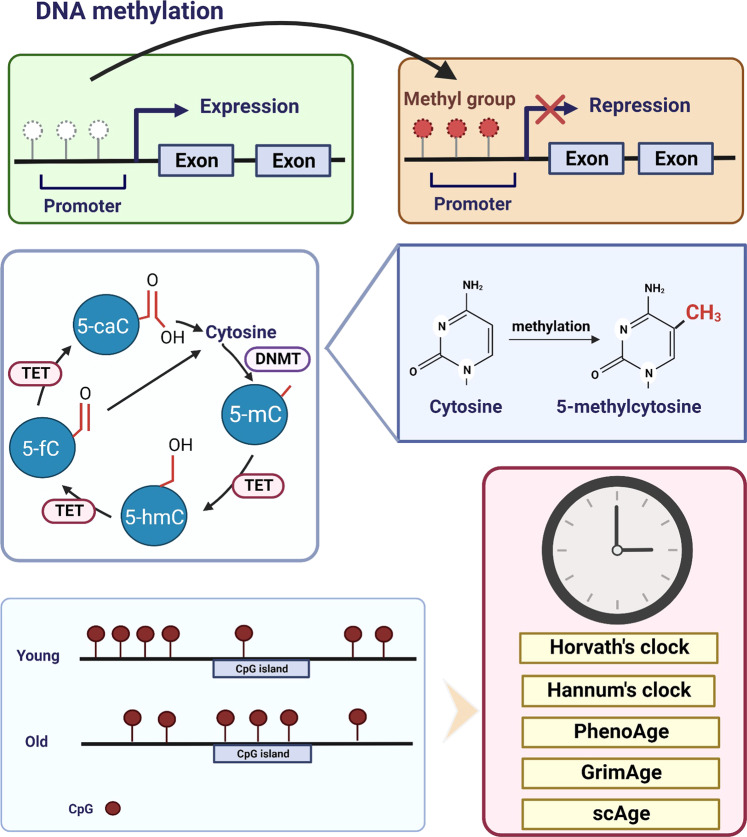
The mechanism of DNA methylation and the epigenetic clock theory of aging. Aging is often marked by global DNA hypomethylation, but hypermethylation also occurs at selective CpG islands. DNA methylation at the promoter of a gene often leads to silencing of that gene. DNA methylation at the 5ʹ cytosine of CpG results in 5-methylcytosine (5-mC). The methylation of DNA is mediated by DNMTs whereas the methyl group on DNA is removed by TET enzymes. TET enzymes oxidize 5-mC to generate 5-mC derivatives, including 5-hydroxymethylcytosine (5-hmC), 5-formylcytosine (5-fC), and 5-carboxylcytosine (5-caC), in mammalian cells. Age estimators, such as Horvath’s clock, Hannum’s clock, PhenoAge, GrimAge and single-cell age clock (scAge) are based on DNA methylation changes in the genome

DNA methylation occurs at the cytosines in CpG dinucleotides to form 5-methylcytosine (5-mC), and 60%-90% of CpG sites in the mammalian genome are methylated (Fig. 3). The genome is generally hypomethylated during aging. Consistent with this, genes in energy metabolism and oxidative-stress resistance show higher expression in skeletal muscle of aged individuals.^32,33^

DNA methyltransferases (DNMTs), namely DNMT1, DNMT3A, and DNMT3B, add methyl groups to nucleotides, resulting in gene silencing.^34,35^ The expression of DNMT1 decreases with age, resulting in a reduced DNA methylation level. DNMT1 mutants that cause the degeneration of selective central and peripheral neurons have been shown to translocate to the cytoplasm and form aggresomes while failing to bind to heterochromatin.^36^ In contrast, the expression of DNMT3A and DNMT3B increases with age, and contributes to de novo methylation of CpG islands in mammalian cells, increases with age.^37,38^ DNA methylation can be removed by ten-eleven translocation (TET) enzymes.^39^ In clinical research of aged patients, mutations of TET2 or DNMT3A increase the expression of pro-inflammatory cytokines and chronic inflammation, which is associated with conventional cardiovascular disease (CVD).^40^

### DNA methylation shift

DNA methylation generally decreases with age in certain human and mouse tissues or cell cultures.^13,41,42,43^ Compared with newborns, whole-genome DNA methylation in CD4^+^ T cells of individuals over 100 years old has been shown to be decreased.^41^ The decrease of 5-mC from young to old mice is also observed in various organs, such as the brain, liver, and small intestinal mucosa, and the loss of 5-mC impairs the physiological function of cells in old mice.^13^ However, there is no notable shift in the genome-wide methylation level during aging in other human cell types, such as cells of the epidermis, liver, and heart, or some rat tissues, such as blood and kidney. These differences in DNA methylation may be due to tissue specificity or different detection techniques. On the other hand, many genes tend to be hypermethylated at CpG islands with aging (Fig. 3).^44–46^ A large meta-analysis of aging-related CpG islands demonstrated that hypermethylation of CpG islands is conserved across 59 tissues, including blood, liver, muscle, skin, brain, and cortex, derived from 128 mammalian species.^47^

Moreover, there are CpG sites that have increased variability in methylation with age, which are called age-associated variably methylated positions (aVMPs).^44,48^ Researchers first identified aVMPs in twin studies, in which older monozygotic twins exhibit a higher level of methylation variation in the overall content of 5-mC than younger twins, meaning that the methylation variation increases with age.^48^ The increased variation in aVMP methylation is associated with the downregulation of the expression of pentose metabolism genes, including *PYGL*, *TALDO1*, and *PGD.*^49^ Apart from aVMPs, there are also specific CpG sites, named age-associated differentially methylated positions (aDMPs).^50^ The methylation rate of aDMPs decreases with age in 6 mammalian species, including human beings, mice, dogs, naked mole rats, rhesus macaques, and humpback whales.^43^ Thus, the DNA methylation shift is associated with different CpG sites, including aVMPs and aDMPs, which can be measured to assess epigenetic age.

### Epigenetic clock

The level of CpG site methylation with age is a reliable biomarker to predict chronological age. Researchers have developed age estimators called epigenetic clocks based on these mammalian DNA methylation levels. Epigenetic clocks use machine learning methods and are based on a set of CpG sites, whose DNA methylation states are consistent in multiple cells, tissues, or organs to predict the chronological age.^51^

The earliest model can estimate the age of a person and predict the risk of aging-related diseases, but shows low precision, only explaining 73% of age variance with a prediction error of 5.2 years.^51^ Since then, multiple epigenetic clocks have been reported with higher accuracy, precision, and broader application prospects in aging research.^52,53^ Among them, the first-generation clocks are Horvath’s epigenetic clock and Hannum’s epigenetic clock.^54,55^ Horvath’s epigenetic clock is a multi-tissue predictor based on 353 CpG sites to estimate the age of most tissues and cell types and is widely used in aging and cancer research.^16^ Hannum’s epigenetic clock can measure and compare human aging rates and provides a quantitative readout for aging-related diseases using 71 CpG markers from the DNA of blood.^52^ Based on Horvath’s pan-tissue clock, the DNAge™ algorithm is developed to compare the chronological age of young and aged muscles.^56^ Later, the second-generation clocks, including PhenoAge and GrimAge, introduced morbidity and mortality into the model, improving accuracy over the first generation.^53,55^ PhenoAge takes into account the role of multiple clinical biomarkers and can predict 10-year and 20-year mortality.^53^ GrimAge is based on 12 plasma proteins and smoking pack-years, and is a more predictive epigenetic clock for identifying clinical phenotypes.^55^ Notably, a recent study built up a single-cell age clock (scAge), which exhibits the epigenetic age using single-cell methylation data.^57^ ScAge is not only able to epigenetically differentiate “young” and “old” cells in heterogeneous tissues, but also predicts the chronological age of the tissues in mice.^57^ In summary, different epigenetic clocks have been developed for the prediction of the chronological age, which can be used to assess the efficacy of intervention methods for aging and to advance precision medicine.

### Histone modification

**Fig. 4:**
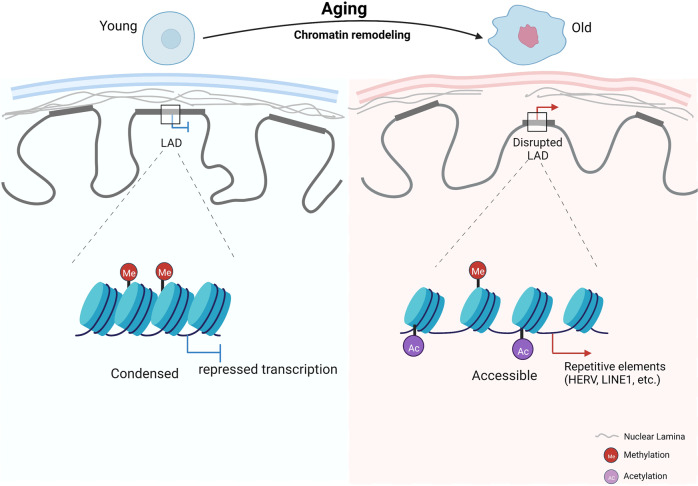
Chromatin structural remodeling during aging. A general loss of heterochromatin and detachment of lamina-associated domain (LAD) structures from the nuclear lamina occur during this process. Higher-order chromatin structure alterations during aging are accompanied by the redistribution of various histone modifications, including histone methylation (H3K4me3, H3K27me3, H3K36me3, H3K9me3) and acetylation (H3K9ac, H3K56ac, H4K16ac, H3K18ac). This leads to reactivation of repeating sequences and dysregulated gene expression due to aberrant chromatin accessibility

Post-translational modifications of histones can activate or silence gene expression and regulate the aging process. The types of histone modifications include methylation, acetylation, phosphorylation, ubiquitination, ADP ribosylation, and others.^58^ Among these modifications, methylation, and acetylation at lysine residues are the most widely studied and are known to affect the aging process. In vivo and in vitro studies report global changes in H3K9me3, H4K20me3, H3K27me3, and H3K9ac levels during aging.^59^ Several enzymes are involved in the regulation of histone methylation and acetylation. Histone methyltransferases (HMTs) and histone demethylases (HDMs) play opposite roles in regulating histone methylation, and histone acetyltransferases (HATs) and histone deacetylases (HDACs) antagonistically regulate histone acetylation (Fig. 4).

### Histone methylation

Previous studies have shown that H3K4me3, a marker associated with active transcription, plays an important role in determining aging and lifespan by regulating the expression of aging-related genes.^60,61^ With aging in yeast, H3K4me3 accumulates in non-promoter regions and ribosomal DNA (rDNA), leading to the loss of rDNA heterochromatin along with an increase in genome-wide pervasive transcription.^62,63^ Studies in *C. elegans* somatic cells have also shown increased enrichment of H3K4me3 in promoter regions of senescence-related genes, and that this dynamics often occurs in regions with relatively low H3K4me3 markers,^64^ while down-regulation of the ASH-2 trithorax complex leads to H3K4me3 deficiency and lifespan extension.^15^ Consistently, ROS stimulation in *C. elegans* juveniles leads to an overall decrease in the H3K4me3 level and enhances their longevity.^65^ In a mouse model of Alzheimer’s disease (AD), the level of H3K4me3 and its catalyzing enzymes increases in the prefrontal cortex, a crucial brain region impaired in AD, and treating these mice with an inhibitor of H3K4 HMTs promotes the recovery of prefrontal cortex functions.^66^ The H3K4me3 level has also been shown to increase with age in mouse hematopoietic stem cells (HSCs).^67^ In contrast, a recent study using physiologically aged human HSCs demonstrated that aging is associated with reduced H3K4me3, H3K4me1, and H3K27ac.^68^ Neurons in aged (>60 years) human prefrontal cortex exhibit loss of H3K4me3 at 556 genes and gain of H3K4me3 at 101 genes compared to young (<1 year) neurons.^69^ Thus, H3K4me3 is related to aging in different species, although its influence on aging is context-dependent and requires further investigation.

H3K27me3 is generally associated with gene silencing and compacted heterochromatin.^70^ Earlier studies suggested a global loss of H3K27me3 in aged *C. elegans* and prematurely aged cells from Hutchinson-Guildford progeroid syndrome (HGPS) patients,^71^ while in killifish and mouse brains, global H3K27me3 increases with age.^72^ In *C. elegans*, the effect of the H3K27me3 demethylase UTX-1 on lifespan seems paradoxical, as both UTX-1 knockdown and overexpression in neurons and the intestine have been shown to extend lifespan.^73–75^ The conserved histone lysine demethylases jmjd-1.2/PHF8 and jmjd-3.1/JMJD3 could work as positive regulators of lifespan in response to mitochondrial dysfunction across different species, suggesting that increased levels of H3K27me3 in genes involved in the mitochondrial unfolded protein response (UPRmt) are detrimental to lifespan.^76^ Determining the locus- and cell-type-specific roles of H3K27me3 in lifespan regulation will be the key to unraveling the impact of H3K27me3-modifying enzymes on aging.

H3K36me3 and H3K9me3 also play important roles in the aging process. In both *S. cerevisiae* and *C. elegans*, deficiency of H3K36me3 is associated with a shorter lifespan. Consistently, the loss of H3K36me3 demethylase extends the lifespan of *S. cerevisiae.*^77^ Similarly, loss of H3K9me3 in the adult *Drosophila* midgut leads to intestinal stem cell aging.^78^ Interestingly, in aged somatic tissues of *C. elegans*, the global H3K9me3 level increases at heterochromatic regions in the distal arms of chromosomes, but decreases in euchromatic central regions of autosomes.^79^ In aged *Drosophila*, H3K9me3 and HP1 signals on chromosomes are significantly reduced compared with those in young flies, and overexpression of HP1 extends lifespan.^80^ Additionally, diminished levels of H3K9me3 and HP1 were identified in mesenchymal stem cells (MSCs) bearing pathogenic mutations of HGPS or Werner Syndrome (WS), another human disease with accelerated aging.^81–83^ The expression of the H3K9me3 methyltransferase SUV39H1 is decreased during the aging of both human and mouse HSCs,^84^ leading to a global reduction in H3K9 trimethylation and perturbed heterochromatin function.^84^ Treatment of Werner syndrome (WS)-specific MSCs with vitamin C, gallic acid (GA), or low-dose chloroquine (CQ) ameliorates a range of senescent phenotypes, promotes cell self-renewal, and upregulates levels of heterochromatin-associated marks, including H3K9me3.^85–87^ Thus, a reduction or redistribution of H3K9me3 is observed across different species with aging, although this trend is also tissue and cell-type dependent.^88,89^

### Histone acetylation

Unlike histone methylation, the relationship between global histone acetylation and longevity is better understood. Histone acetylation is mediated by lysine acetyltransferases and is increased in active gene regions. HDACs are considered to function as corepressors and, together with HATs, play a critical role in longevity. Sirtuins are class III HDAC that enhance genome stability and regulate the deacetylation of lysine residues in an NAD^+^ level-dependent manner.^90,91^ Among the sirtuin family members, SIRT1 has been reported to decrease with age in various tissues of humans and mice, such as the liver, heart, kidney, brain, and lung.^92,93^ SIRT6 functions as an NAD^+^-dependent H3K9 deacetylase that modulates telomeric chromatin, and its overexpression contributes to the longevity of rat and human nucleus pulposus cells via inhibiting senescence.^94^ On the other hand, the HDAC class II family member HDAC4 was reported to be polyubiquitylated and degraded during all types of senescence. HDAC4 selectively binds to and monitors H3K27ac levels at specific enhancers and super-enhancers, such as enhancers of *AKR1E2* and *VEGFC*. Treatment with the inhibitor of the HAT P300 could rescue senescence in HDAC4-depleted cells, suggesting a potential antagonistic effect between HDAC4 and P300.^95^ In IMR90 cells, P300, which promotes the formation of active enhancer elements in the non-coding genome, significantly increases the levels of H3K122ac and H3K27ac at the proximal senescence-specific gene promoters and is confirmed to be a primary driver of the senescent phenotypes, and depletion of p300 alone is sufficient to downregulate senescence genes and delay replicative senescence.^96^ CBP-1, the homolog of mammalian acetyltransferase CBP/p300 in *C. elegans*, is an essential regulator of the UPRmt and mediates H3K27ac and H3K18ac upon mitochondrial stress. Knockdown of CBP-1 decreases the lifespan of the worm in an HSF-1-dependent manner.^97,98^ Interestingly, abundant H3K4me1 marks are displayed in replicatively senescent IMR90 cells at the senescence-associated secretory phenotype (SASP)-associated super-enhancer loci, which also show substantial H3K27ac marks and BRD4 binding.^96,99^ Recent studies also demonstrated that aging-mediated changes in H3K27ac and H3K9ac in the human cerebral cortex are associated with AD.^100,101^

Owing to the antagonistic actions of HATs and HDACs, it is not surprising that HATs have also been implicated in aging. The level of H3K14ac in the brains of aging mice can regulate the expression of aging-related synaptic plasticity genes, and the H3K9me3/H3K14ac bivalent marks are significantly decreased in old mouse hepatocytes.^102,103^ Inactivation of KAT7 decreases histone H3K14ac and alleviates human mesenchymal precursor cell (hMPC) senescence.^104^ H3K18ac and H3K56ac are negative markers of senescence in *Drosophila* and yeast.^105,106^ Although the mechanism of action is different, knockdown of H3K56ac, Hst3, and Hst4-related HDAC-encoding genes during yeast aging shortens the lifespan.^107^ In aged yeast, the H3K56ac level decreases while the H4K16ac level increases, leading to the silencing of telomeric repeats.^108^ Interestingly, H4K16ac may also be involved in brain aging and AD progression. Normal aging leads to H4K16ac enrichment, while H4K16ac in the proximity of genes linked to aging and AD is dramatically reduced in AD.^109^ Additionally, dysregulation of H4K12ac leads to aging-related memory impairment, suggesting that it may serve as a critical signal of memory formation.^110^ By administering the HDACi suberoylanilide hydroxamic acid (SAHA) to aged mice, the acetylation deficit of H4K12 could be rescued in neurons.^111^ Thus, substantial changes in histone acetylation occur during aging and aging-related diseases, and understanding its regulatory mechanisms may provide new insight into the development of aging-intervention strategies.

### Histone phosphorylation and ubiquitination

In addition to histone methylation and acetylation, histone phosphorylation and ubiquitination have also been shown to be associated with aging, and in some cases through crosstalk with other histone marks. For example, the effect of histone ubiquitination on DNA damage accumulation can induce premature neuronal aging.^112^ H3S28A mutants, which depletes H3S28 phosphorylation but also reduces H3K27 methylation to prevent by compromising the activity of its methyltransferase complex in *Drosophila*, prolong lifespan and improve resistance against starvation and paraquat-induced oxidative stress.^113,114^ However, the correlation between these modifications and aging is less clear, and more research is needed to refine the mechanisms in the future.

### Chromatin remodeling

Chromatin is a flexible and dynamic structure composed of DNA and histones that can exist as heterochromatin or euchromatin. The basic unit of chromatin is the nucleosome core particle, encapsulated in a histone octamer consisting of a central H3-H4 tetramer flanked by two H2A-H2B dimers.^115^ Chromatin remodeling is defined as a series of genome-wide changes in the nuclear architecture that can be recognized at the level of specific chromosomes or chromosome domains, such as centromeres. Significant chromatin structural remodeling has been identified during cellular senescence, from histone component and modification changes to alterations of the chromatin compartments and topologically associating domains (TADs).^83,116,117^ Global canonical histone loss is regarded as a common feature of aging from yeast to humans.^108,118,119^ Overexpression of histone H3/H4 in yeast extends the lifespan, suggesting that an increased pool of free histones promotes survival during aging by facilitating nucleosome exchange and post-transcriptional chromatin repackaging.^106^ Genome-wide profiling of the core histone H3 occupancy in primary cultures of aging male mouse tissues and neural stem cells (NSCs) reveals local changes in H3 occupancy as tissues and cells age, even though the H3 level remains relatively stable.^120^ Reversible phosphorylation of serine and threonine residues in the C-terminal tail of H1 histones is responsible for regulating the H1 stacking behavior. Individuals with mutations deleting these residues in one of the histone H1 isoforms show a progeria phenotype, and their fibroblasts exhibit more nucleoid relaxation, less condensed chromosomes, and higher nucleolar instability (Fig. 4).^121^

In eukaryotes, histone-modifying enzymes and ATP-dependent chromatin-remodeling complexes are the two main factors of the chromatin-remodeling process.^122^ Modified histones may induce conformational changes in nucleosomes. Restoration of acetyl coenzyme A (acetyl-CoA) production through nutrient supplementation (citrate, acetate, pyruvate, and glucose) could strongly attenuate chromatin reorganization and diminish the extended lifespan of worms under mitochondrial stress conditions.^123,124^ In mice, aged MSCs show significantly decreased levels of total histone H3-H4 acetylation and an increased abundance of H3K27me3 across the gene body, resulting in a lower transcriptional rate and the loss of chromatin accessibility compared with young MSCs. Restoring cytoplasmic acetyl-CoA levels in aged MSCs can remodel chromatin structure and rejuvenate these cells.^125^ As H3K9me2 levels decrease, the nuclear peripheral heterochromatin loses its anchor to the nuclear lamina and moves toward the nuclear interior.^126^ In specific regions during aging, H3K9me2 switches to H3K9me3, another repressive mark but not enriched with direct contacts with the nuclear lamina; this may reflect aging-associated changes in subnuclear location of peripheral chromatin and associate with shortened lifespan in aged *C. elegans* somatic tissues.^79^ Interestingly, histone deacetylase or methyltransferase inhibitors alter histone modifications in ways that predominantly increase euchromatin or decrease heterochromatin.^127^ These results suggest that chromatin remodeling is largely related to the level of histone post-translational modifications. In addition, deletion of autophagy-related 7 (Atg7) leads to disordered nucleosome assembly in mouse CD11b^+^Ly6G^-^ bone marrow cells, resulting in cellular senescence.^128^ Promoters of the conserved transcriptional and phenotypic responses to defects in chromatin structure genes and are sensitive to histone dosage. Reducing nucleosome occupancy at these promoters by deleting *HHT1-HHF1* allows transcriptional activation induced by the stress-responsive transcription factors Msn2 and Gis1, and thus, responses induced by moderate chromatin architectural defects promote longevity.^129^

ATP-dependent chromatin-remodeling complexes can be divided into the SWI/SNF, ISWI, CHD, and INO80 families. SWI/SNF is required for the activation of nutrient-responsive genes, and the destruction of this complex impairs the ability of cells to adapt to their environment. SWI/SNF also regulates transcription by remodeling chromatin and promoting a more open chromatin configuration.^130^ In vitro, the BRM-SWI/SNF complex is required to promote co-expression of the telomere-binding proteins TRF1 and TRF2, which are essential for maintaining telomere length and structure in human fibroblasts and cervical cancer cells, contributing to the development of longevity-related functions.^131^ SWI plays a role in regulating aging during adulthood, and the absence of SWI shortens the lifespan of nematodes.^132^ Among CHD chromatin remodelers, the role of NuRD has been widely reported, and disruption of the NuRD complex may compromise the epigenetic composition of histones and the higher-order structure of chromatin, making the NuRD complex more susceptible to the influence of genotoxic stress.^133^ *BAZ1A* encodes an accessory subunit of the ATP-dependent chromatin-remodeling complex that regulates cellular senescence in cancer and normal cells.^134^ Inhibition of the chromatin-remodeling factor SMARCA4 is able to prevent aging-dependent dopaminergic degeneration and shortening of lifespan caused by α-synuclein and LRRK2 in *Drosophila* PD models.^135^

Chromatin accessibility states and the expression programs of aging-related genes are positively correlated during aging. Two types of chromatin regions with regular changes in their accessibility during aging are increased accessibility regions (IARs) and decreasing accessibility regions (DARs). IARs mainly exist in genes related to the occurrence and development of aging, whereas DARs mainly exist in genes related to functional decline caused by aging.^136,137^ The chromatin in human cells is spatially segregated into two compartments, compartment A and compartment B, and chromatin in the same compartment should have more frequent interactions, as revealed by Hi-C analysis.^138^ The disruption of higher-order chromatin structure and the separation of heterochromatin from the nuclear membrane are observed during cellular senescence and aging.^63,83,130,139–141^ A hierarchy of integrated structural state changes has been characterized through large-scale epigenomic analyses of isogenic young, senescent, and progeroid hMPCs, manifested as heterochromatin loss in repressive compartments, euchromatin weakening in active compartments, switching in interfacing topological compartments, and increasing epigenetic entropy. Nuclear lamina dysfunction results in the derepression of constitutive heterochromatic regions in repressive LAD structures marked by H3K9 methylations. Diminished histone markers such as H3K27me3 and facultative heterochromatin disruption contributed to an overall increase in epigenetic instability and ectopic expression of lineage restricted genes, SASP genes and repetitive elements.^83,142–145^ Loss of heterochromatin leads to a global increase in transcription and intercellular transcriptional heterogeneity, which is reported to be associated with cellular senescence and the onset of aging-related diseases.^81,137,143,146,147^ Higher resolution data show that chromosome compartments are partitioned into TADs, which are more basic domains with high interaction frequency therein and relatively isolated from neighbor regions. Below the scale of TADs, long-range chromatin looping interactions are insulated by TAD boundaries.^148^ CTCF, a highly conserved architectural protein with 11 zinc fingers, functions as a barrier to inhibit heterochromatin spreading,^149^ and it has been shown to be reduced during aging in various models and plays an important role in chromatin remodeling.^150^ Consistent with this, high levels of CTCF in proliferating fibroblasts promote p16^INK4a^ silencing.^151^

### RNA modification

**Fig. 5:**
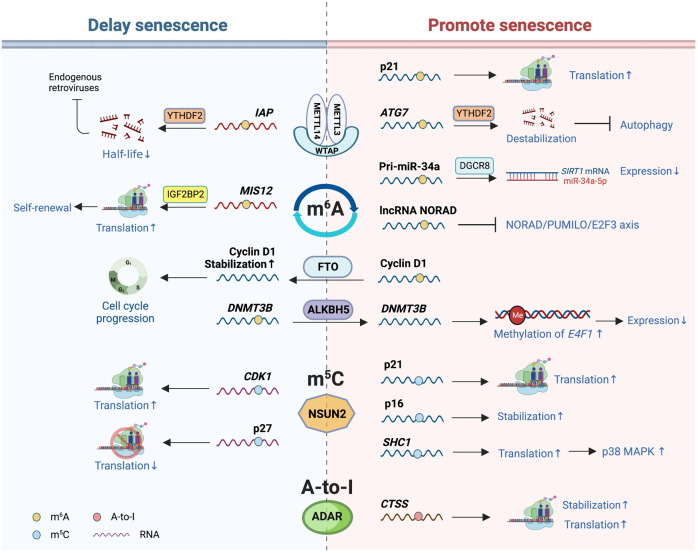
RNA modifications involved in senescence. RNA modifications that have been revealed to be associated with senescence or aging mainly include m^6^A modification, m^5^C modification, and adenosine-to-inosine (A-to-I) editing. The m^6^A modification is regulated by factors including the writer RNA methyltransferases complex, the erasers FTO and ALKBH5, and the reader YTHDF2, whose effects on senescence are complex based on different substrates. Under different stress conditions, m^5^C modification mediated by NSUN2 plays opposite roles in senescence, retarding replicative senescence and accelerating oxidative-induced senescence. The A-to-I RNA editing catalyzed by the ADAR family mainly exists in the central nervous system, and its relationship to neurodegenerative diseases has been demonstrated

More than 170 types of RNA modifications have been discovered thus far that regulate gene expression at the epitranscriptomic level.^152^ Although few studies have directly revealed the relationship between RNA modifications and organismal aging, accumulating data show the essential roles of these post-transcriptional regulatory mechanisms in the cellular senescence process, one of the critical causes for aging and aging-related diseases (Fig. 5).

### m^6^A modification

As one of the most extensively studied mRNA modifications in mammalian cells, m^6^A has been demonstrated to be involved in cellular senescence. m^6^A is regulated by writer, reader, and eraser proteins.^152,153^ The multi-subunit writer RNA methyltransferases (MTases) are assembled mainly by methyltransferase like 3 (METTL3), METTL14, and Wilms tumor 1 associating protein (WTAP).^154^ The first reported m^6^A modification involved in senescence is methylation at the 3ʹ-UTR of the *CDKN1A* mRNAs by a METTL3/14 heterodimer, which facilitates p21 translation. Consistently, the expression of METTL3/14 and p21 is enhanced in oxidative-stress-induced senescence.^17^ Recently, *ATG7* mRNAs with METTL3-dependent m^6^A were found to be destabilized by the reader YTH N6-methyladenosine RNA-binding protein 2 (YTHDF2), which promotes senescence instead of autophagy in fibroblast-like synoviocytes and leads to the progression of osteoarthritis.^155^ METTL14 also catalyzes the m^6^A modification affecting miRNAs associated with senescence. For example, TNF-α-induced METTL14 overexpression leads to increased production of miR-34a-5p from m A-modified primary transcript. miR-34a-5p promotes celluar senescence by targeting Sirtuin-1 (SIRT1) in nucleus pulposus cells (NPCs) of patients with intervertebral disc degeneration (IVDD), one of the most prevalent degenerative diseases.^156^ More recently, the regulator WTAP, which functions to translocate METTL3/14 dimers to nuclear speckles,^154^ has also been demonstrated to be associated with IVDD. Increased WTAP in senescent NPCs enhances the level of m^6^A in the lncRNA NORAD, contributing to the disruption of the NORAD/PUMILO/E2F3 axis and accelerating senescence.^157^

METTL3/14-mediated m^6^A modification has also been reported to inhibit senescence in some cases. METTL3/14 levels are reduced in LMNA mutant-induced prematurely aged human HGPS cells and senescent fibroblasts, and METTL14 overexpression delays cellular senescence.^158^ The interaction of Lamin A and METTL3/14 protects the latter from proteasome-mediated degradation to maintain sufficient m^6^A levels in normal cells.^158^ Moreover, METTL3-mediated m^6^A modification of *MIS12* mRNAs positively regulates their stabilization by recruiting IGF2BP2, and in young hMSCs, MIS12 facilitates their self-renewal and alleviates cellular senescence. In HGPS and WS hMSCs, cellular models of premature aging, the downregulation of MIS12 is detected at both the mRNA and protein levels.^159^ Knockdown of DNMT2 in mouse embryonic fibroblasts (MEFs) reduces the m^6^A level and accelerates senescence.^160^ Thus, in addition to RNA MTases, DNMT2 participates in senescence regulation by affecting the m^6^A level. Sulforaphane-mediated cycle arrest and senescence in breast cancer cells are also accompanied by downregulated global m6A levels of mRNAs; however, the underlying mechanism is unclear.^161^

Similar to these m^6^A writers, the main erasers of m^6^A, i.e., fat mass and obesity-associated protein (FTO) and alkB homolog 5 (ALKBH5), are also involved in aging. For example, the expression of FTO declines with ovarian aging, followed by increased m^6^A levels in old human granulosa cells.^162^ Furthermore, FTO is crucial for the progression of the G1 phase of the cell cycle by removing m^6^A from the cyclin D1 mRNAs and stabilizing them.^163^ The ALKBH5 level is increased during IVDD and NPC senescence, and it removes m^6^A from the *DNMT3B* mRNAs, which limits the expression of the transcription factor E4F1 by methylating CpG islands at its promoter region and accelerates NPC senescence.^164^

To execute the function of m^6^A, reader proteins are needed, which include YTHDC family members, YTHDF family members, the eukaryotic translation initiation factor eIF3, the insulin-like growth factor 2 mRNA- binding proteins (IGF2BP1/2/3), and the fragile X retardation protein (FMRP).^165^ Several studies have indicated that the YTHDF family plays an important role in cellular senescence by destabilizing targeted mRNAs. In mouse embryonic stem cells (ESCs), m^6^A-modified intracisternal A-particle (*IAP*) mRNAs recruit YTHDFs to shorten their half-life, repressing endogenous retroviruses (ERVs).^166^ Conversely, the accumulation of *IAP* mRNAs after deletion of YTHDFs leads to high ERV activity,^166^ resulting in senescence and diseases.^167^ The well-known senolytic therapy (discussed in the next chapter) of the combination of dasatinib and quercetin can reduce the lipopolysaccharide (LPS)-induced SASP by upregulating YTHDF2, followed by destabilization of *MAP2K4* and *MAP4K4* mRNAs in human umbilical vein endothelial cells (HUVECs).^168^

In addition to cellular senescence, the role of m^6^A methylation in organs or organismal aging remains elusive. In brain aging and neurodegenerative disease models, dysregulation of m A and related regulatory proteins was indicated but the findings varied. For example, a tendency toward an overall increase in m^6^A methylation was observed in the cortex and hippocampus of the APP/PS1 transgenic mouse model for AD.^169^ Yet m^6^A in the 3ʹ UTR of many AD-associated transcripts in the 5XFAD mouse model of AD is downregulated, which is accompanied by an 8% increase and 4% decrease in FTO and METTL3, respectively, compared to wild-type mice, leading to higher Tau toxicity using the AD fly model.^170^

### m^5^C modification

The m^5^C modification of RNAs is also tightly associated with senescence, in which the diverse roles of the tRNA methyltransferase NSUN2 (NOP2/Sun domain family, member 2) depend on the substrates of the m^5^C modification. For example, the 3ʹ-UTR of cyclin‐dependent kinase 1 (*CDK1*) mRNAs and the 5ʹ-UTR of the CDK inhibitor p27 mRNAs are targets of NSUN2, and m^5^C modification facilitates the translation of CDK1 while repressing that of p27, both of which alleviate replicative senescence.^171,172^ Interestingly, NSUN2 shows the opposite function in senescence under oxidative-stress conditions by targeting other mRNAs. NSUN2-mediated m^5^C at A988 in the 3ʹ-UTR of cyclin-dependent kinase inhibitor 2A (*CDKN2A*, i.e., p16) mRNAs stabilizes the mRNAs, contributing to senescence.^173^ H_2_O_2_ treatment leads to upregulation of NSUN2 and Src homology 2 domain-containing (SHC) family proteins in HUVECs with accelerated senescence, whereas knockdown of NSUN2 reduces ROS accumulation and delays senescence. NSUN2 catalyzes m^5^C modification of *SHC1* mRNAs at several sites, which promotes its translation, activates the p38 MAPK pathway, and leads to cell cycle arrest and elevated ROS levels.^174^ Strikingly, NSUN2 and METTL3/14 have synergistic effects on the p21 mRNA methylation induced by oxidative stress. The m^5^C modification induced by NSUN2 facilitates METTL3/14 to catalyze the m^6^A modification at the p21 mRNA 3ʹ-UTR and vice versa.^17^ Furthermore, the RNA-binding protein human antigen R (HuR) facilitates the m^5^C modification at the C106 site of *TERC* to enable telomerase activity and delay cellular senescence.^175^ In summary, these studies identify RNA m^5^C as an epitranscriptomic marker for aging, and more investigation at the genome-wide scale will further reveal the dynamics of the m^5^C landscape and its potential impact on cellular senescence and tissue and organismal aging.

### A-to-I editing

Adenosine to inosine (A-to-I) RNA editing conducted by the adenosine deaminase acting on RNA (ADAR) family occurs most frequently in the central nervous system, and the A-to-I imbalance has been demonstrated to be involved in neurological disorders, metabolic diseases, and other diseases.^176,177^ Insufficient A-to-I editing influences neurodegenerative processes. The editing level at the GluA2 Q/R site in the hippocampal region is lower in AD, which induces altered Ca^2+^ influx and neuron death.^178^ In human endothelial cells under pro-inflammatory conditions, cathepsin S (*CTSS*) mRNAs, encoding a cysteine protease, can be targeted by ADAR1, thereby recruiting HuR to improve its stability and translation. Consistently, the frequency of A-to-I editing on *CTSS* mRNAs is much higher in patients with vascular diseases.^179^

Collectively, these reports indicate that RNA modifications play crucial roles in regulating senescence. However, further studies are needed to uncover more detailed underlying mechanisms and their relationship to organismal aging, which may provide an avenue for developing new treatments for ameliorating senescence.

### Non-coding RNA regulation

**Fig. 6:**
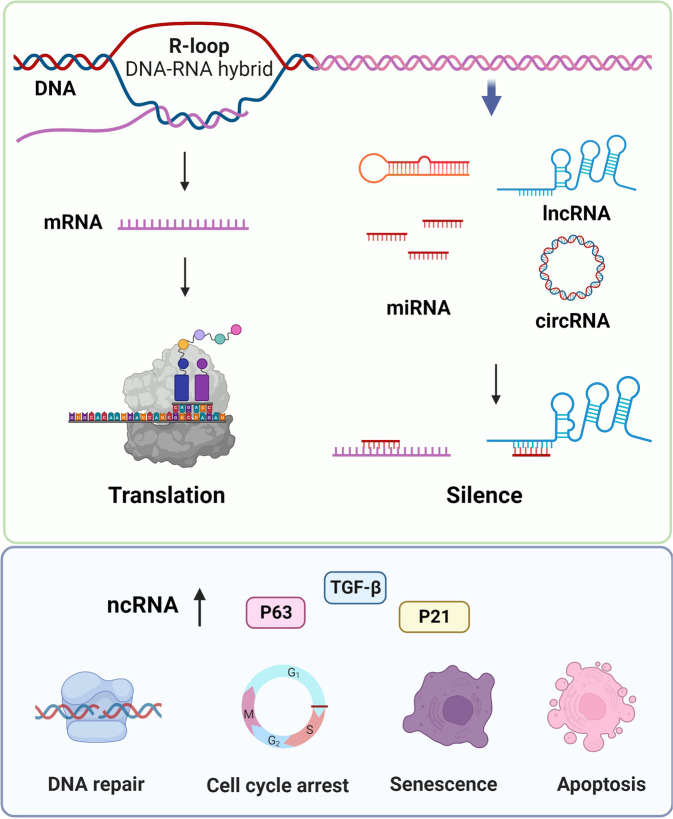
The mechanism of non-coding RNAs regulation during aging. Non-coding RNAs (ncRNAs) include microRNAs (miRNAs), long non-coding RNAs (lncRNAs), R-loop (DNA-RNA hybrids), and circular RNAs (circRNAs). miRNAs bind to mRNAs, lncRNAs or circRNAs to prevent their functions

Research on the molecular mechanisms of cellular aging has mainly focused on protein-coding genes. However, accumulating studies have demonstrated that ncRNAs, which widely regulate gene expression in multiple biological processes at the epigenetic, transcriptional, and post-transcriptional levels, also play a critical role in aging. In recent years, studies of ncRNAs in aging have mainly focused on microRNAs (miRNAs),^180^ long non-coding RNAs (lncRNAs),^181^ R-loops,^182,183^ and circular RNAs (circRNAs)^184^ (Fig. 6).

MiRNAs are small (~22 nucleotides), non-coding and single-stranded RNAs that bind to the 3ʹ-UTR of target mRNAs to degrade these mRNAs or suppress their translation.^185,186^ Using a microarray containing 863 miRNAs, researchers discovered that 64 miRNAs, such as miR-30d, miR-320d and miR-339-5p, are upregulated and 16 miRNAs, such as miR-103, miR-107, miR-24, and miR-130a, are downregulated in long-lived individuals compared to younger individuals.^180^ In addition, the miRNA-p53 pathway can maintain the genomic integrity in long-lived individuals during aging.^180^ The expression of miR-217 increases in late-passage fibroblasts, where miR-217 inhibits DNMT1 expression by targeting its 3′-UTR to induce human skin fibroblast senescence.^187^ The expression of age-associated miRNAs, including miR-130, miR-138, and miR-181a/b, increases in keratinocytes during cellular senescence, and by binding to p63 and Sirtuin-1 mRNAs, these miRNAs affect cell proliferation pathways.^188^

LncRNAs are non-protein-coding RNAs longer than 200 nucleotides. LncRNAs bind to DNA, RNA, and proteins to exert their functions as guides, enhancers, or scaffolds in post-transcriptional and post-translational regulations.^189^ Therefore, lncRNAs have become targets for the treatment of fibrosis in aging. In aged bone marrow mesenchymal stromal cells, the lncRNA NEAT1 promotes CSF1 secretion and enhances osteoclastic differentiation, which may be a therapeutic target for skeletal aging.^190^ The lncRNA APTR accelerates the cell cycle and cell proliferation of primary hepatic stellate cells in mice.^191^ Furthermore, targeting the lncRNA *Firre* by CRISPR/Cas9 delays Ras-induced cellular senescence.^192,193^

Evidence shows that circRNAs play important roles in the modulation of aging and aging-related diseases, such as cardiovascular disorders, diabetes, and neurodegenerative diseases.^194^ As circRNAs are relatively stable, aging-related increases in global circRNA levels are potential diagnostic biomarkers for aging.^195^ R-loops are three-stranded structures composed of a DNA-RNA heteroduplex and a displaced single DNA strand. Although R-loops are often considered as “by-products” of transcription, recent studies have shown that R-loops are important cellular regulators and may contribute to cancer and neurodegeneration.^183,196,197^ For example, deletion of SPT6 extends lncRNA and increases R-loops associated with DNA damage, which ultimately leads to senescence in HeLa cells.^198^ In summary, ncRNAs (miRNAs, lncRNAs, and circRNAs) have been proven to serve as biomarkers in regulating cellular senescence.

### Strategies to alleviate aging

**Fig. 7:**
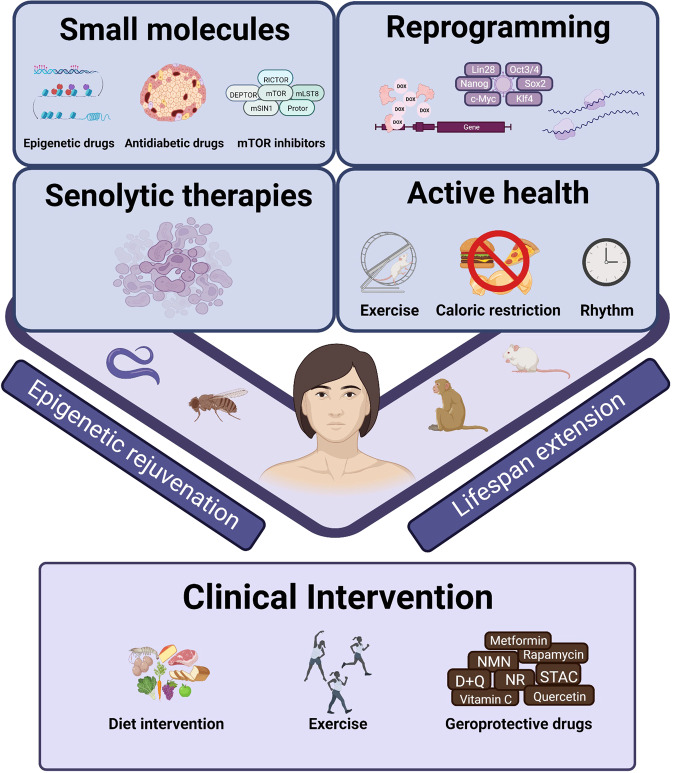
The intervention of aging. Existing intervention strategies aim to alleviate aging in various organisms

Based on the molecular mechanisms underlying cellular senescence and aging, a series of therapeutic strategies, many of which are closely related to epigenetic regulations, have been proposed (Fig. 7). Reprogramming and geroprotective drugs have been developed to interfere with aging, while senolytics aim to remove senescent cells to delay aging. Active health, such as caloric restriction, exercise, and a healthy circadian rhythm, exerts profound influences on multiple organs, systemic circuitries, and whole-body rejuvenation. Moreover, several advanced intervention methods have entered the clinical trial. Below we will discuss all these aging-intervention strategies and their underlying epigenetic mechanisms.

### Small molecule-based therapy

**Fig. 8:**
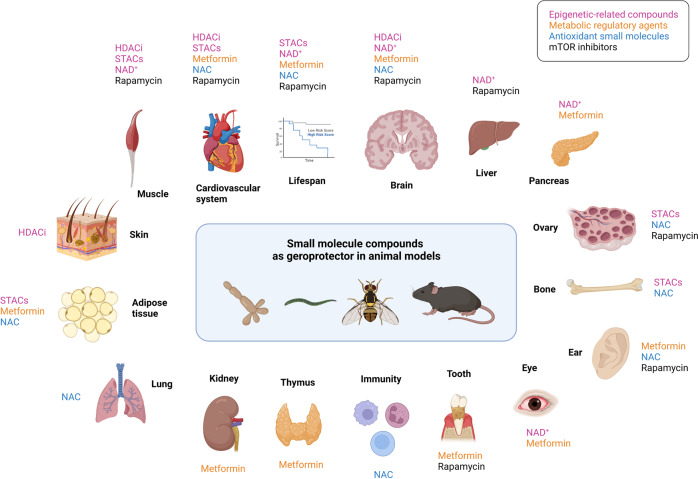
Small molecule compounds as geroprotectors in diverse animal models. A series of small molecule compounds can extend the lifespan or alleviate aging-related phenotypes in different organs. The interventions and corresponding target organs are shown in the diagram

The first class of aging-intervention strategies enumerated here is geroprotective drugs, which include epigenetic-related compounds (e.g., NAD^+^ precursors, sirtuin-activating compounds, and HDAC inhibitors), small molecules with robust anti-diabetic effects (e.g., metformin), mTOR inhibitors (rapamycin), as well as antioxidant chemicals (N-acetyl-l-cysteine) (Fig. 8).

### NAD^+^ precursor

NAD^+^ is a critical redox coenzyme that plays a unique role in aging through DNA repair and epigenetic regulation.^199,200^ The effect of sirtuins on histone deacetylation is highly dependent on NAD^+^, highlighting the indispensable role of NAD^+^ in the epigenetic regulation of aging.^201^ Supplementation with NAD^+^ precursors, such as nicotinamide mononucleotide (NMN), nicotinamide riboside (NR), and nicotinamide (NAM), prevents the decline in NAD^+^ and exhibits beneficial effects against aging and aging-related diseases. NAD^+^ repletion extends the lifespan and delays the accelerated aging in *C. elegans* and *Drosophila melanogaster* models of Werner syndrome.^202^ In mammals, NR supplementation increases mitochondrial function, delays the senescence of NSCs, and increases mouse lifespan.^203^ NR supplementation also increases mitochondrial function and reduces aging-associated amyloidosis in muscle.^204^ In addition, NAD^+^ repletion with either NMN or NR ameliorates aging-associated meibomian gland dysfunction in aged mice.^205^ It also improves cognitive functions in AD mouse models, mainly by rescuing cerebral microvascular endothelial function and neurovascular coupling responses, preventing amyloid-β (Aβ) production in the brain, and reducing DNA damage, neuroinflammation, and apoptosis of hippocampal neurons.^206–208^ NAM improves glucose homeostasis and reduces hepatic steatosis and inflammation.^209^ Thus, boosting the NAD^+^ level appears to be a promising therapeutic strategy to counter aging and aging-associated disorders, although its effects in humans need further clinical studies.

### Sirtuin-activating compound

Activators of the sirtuin family of HDACs, also termed sirtuin-activating compounds (STACs), are another class of epigenetic drugs as potential geroprotectors. Since they were found to promote the lifespan of yeast,^24^ STACs have been demonstrated to extend the longevity of worms, fruit flies, honey bees, and fish.^210^ In mammals, resveratrol, an activator of sirtuin 2, increases insulin sensitivity and motor function and thus improves the health and survival of mice on a high-calorie diet.^211^ Resveratrol is also found to attenuate the aging of adipose stem cells via decreasing the levels of 5-mC in DNA and modulating mitochondrial dynamics.^212^ SRT1720, an activator of sirtuin-1, can attenuate vascular endothelial dysfunction, excessive superoxide production, aging-related metabolic diseases, and inflammation with aging, as well as improve the follicle pool reserve, thereby extending the lifespan and improving the healthspan of mice.^213–215^ SRT2104, another activator of sirtuin-1, preserves bone and muscle mass and extends the survival of male mice on a standard diet.^216^

### HDAC inhibitor

As discussed earlier, histone acetylation is one of the most important patterns of epigenetic regulation during aging. HDAC inhibitors show geroprotective effects mainly through reversing aging-associated deacetylation of chromatin, acetylation of histones near pro-longevity genes, and activating stress resistance and pro-longevity proteins.^217^ Administration of the pan-HDAC inhibitor SAHA rescues the skin phenotype, such as loss of subcutaneous fat, inflammation, and fibrosis, in a mouse model of Cockayne syndrome (CS), a hereditary form of premature aging.^218^ ITF2357 (givinostat) suppresses aging-induced diastolic dysfunction in normotensive mice.^219^ Another HDAC inhibitor, butyrate protects against aging-related muscle atrophy in mice.^220^ HDAC inhibitors also exhibit beneficial effects in neurodegenerative disorders by modulating chromatin-mediated neuroplasticity and improving learning consolidation.^221,222^ Since sirtuins are also a class of HDACs, the mechanism by which both STACs and HDAC inhibitors can delay aging remains to be further investigated.

### Metformin

Metformin is an anti-diabetic drug and one of the most attractive geroprotective compounds, and it functions through extensive epigenetic regulation. Metformin retards aging in *C. elegans* by altering the ratio of S-adenosylmethionine (SAMe)/S-adenosylhomocysteine (SAH), which may affect histone methylation.^223^ In a spatial restraint stress mouse model, metformin exerts antidepressant effects by increasing the DNA 5-hmC modification level of the *Bdnf* gene.^224^ Metformin treatment increases the microRNA-processing protein DICER1 in mice and humans and thus modifies the profile of microRNAs associated with senescence and aging.^225^ Administration of metformin also alleviates the senescence of dental pulp stem cells through AMPK/mTOR signaling pathway-mediated downregulation of miR-34a-3p and upregulation of CAB39.^226^ Strikingly, there is evidence that metformin intervention improves the lifespan and healthspan of mice even when the administration starts at middle age (12 months)^227^ or old age (20–24 months),^228–230^ and the effect is enhanced when it starts earlier.^231^ In female SHR mice, however, metformin administration starting at the age of 3 months increases the mean lifespan by 14%, whereas the increase is only 6% when it starts at the age of 9 months, and there is no increase when it starts at the age of 15 months. Consistent with this, the lifespan extension effect of metformin is not seen in male rats^232^ or aged female mice.^233^ Nevertheless, metformin relieves many aging-related diseases in rodent models, including cognitive impairment and neurodegeneration,^229,234–237^ depression,^238^ chronic kidney disease,^239^ thymus degeneration,^240^ aging-related cataract,^228^ aging-related hearing loss,^241^ mitochondrial dysfunction in aged hearts,^230^ adipose tissue senescence and metabolic abnormalities,^242,243^ and aging-related developmental and metabolic phenotypes.^244^

### Rapamycin

Rapamycin, an approved immunosuppressant in solid organ transplantation, also shows potential to intervene with aging. Rapamycin extends the median and maximum lifespan of both male and female mice in a dose-dependent manner through multiple mechanisms,^245–247^ including attenuating aging-related DNA methylation changes in the hippocampus to affect brain aging,^248^ slowing the aging epigenetic signatures in mouse livers, and ameliorating a series of aging-related diseases including cardiovascular dysfunction,^249,250^ neurodegeneration,^251–254^ skeletal muscle aging,^255,256^ ovarian aging,^257,258^ aging-related hearing loss,^259,260^ and aging-associated periodontitis.^261,262^ However, prolonged rapamycin administration is reported to induce muscle insulin resistance in rats, which might increase the incidence of diabetes.^263^ Considering the immunosuppression and NSC suppression effects of rapamycin,^264^ its application as a geroprotector should be assessed further.

### N-acetyl-l-cysteine

N-acetyl-l-cysteine (NAC) is an antioxidant with a prominent influence on epigenetic regulation. It delays oocyte aging in mice by increasing the expression of sirtuins.^265^ Similarly, NAC attenuates aging-related oxidative damage and neurodegeneration in rat brains by upregulating sirtuin-1 and downregulating several SASP factors (TNF-α, IL-1β, IL-6).^266^ In addition, NAC extends the lifespan of mice^267^ and ameliorates a series of aging-related diseases in rodents, such as AD,^268,269^ aortic fibrosis,^270^ immunosenescence,^271^ oxidative stress and senescence in the lung,^272^ bone loss in ovariectomized mice,^273^ adipose tissue senescence and metabolic abnormalities,^243^ and aging-related hearing loss.^274^

### Other geroprotective drugs

Many other drugs also show geroprotective effects, including anti-diabetic drugs (sodium-glucose cotransporter-2 inhibitors,^275^ acarbose,^276–278^) natural compounds (gallic acid,^86^ quercetin,^279,280^) antioxidant molecules (vitamin C,^85,281^ methylene blue,^282^) antihypertensive drugs (angiotensin-converting enzyme inhibitors and angiotensin receptor blockers),^283,284^ chloroquine,^87,285^ aspirin,^286^ uridine,^287^ and so on.

### Reprogramming strategy

Reprogramming somatic cells with Yamanaka factors (Oct3/4, Sox2, Klf4, and c-Myc; OSKM) reverses cell fate and finally generates induced pluripotent stem cells (iPSCs), which possess the characteristics of ESCs.^288,289^ A classic strategy for combating aging comes from the generation of iPSCs. The durable expression of OSKM leads to widespread chromatin remodeling,^290^ and interestingly, some aged somatic cells can be reprogrammed to exhibit a youthful state. Ectopic expression of OSK without c-Myc restores the young patterns of DNA methylation and transcriptomes in mouse retinal ganglion cells, which can ameliorate vision problems in glaucomatous in aged mice. The DNA demethylation induced by OSK expression is confirmed to be necessary for the rejuvenation process of retinal ganglion cells.^291^

Although long-term reprogramming rejuvenates aged cells to varying degrees, some of the aged somatic cells will be fully reversed to iPSCs, which makes it impossible to be used to delay aging in vivo due to the teratoma-forming ability of iPSCs.^292^ Notably, transient reprogramming, which allows the expression of reprogramming factors in a certain period of time, also exerts a rejuvenating effect on aged somatic cells without altering the original cell identities.^293,294^ Transfecting aged human fibroblasts, chondrocytes, and endothelial cells with mRNAs expressing OSKMLN (OSKM, LIN28 and NANOG) rejuvenates host cells and significantly reverses the epigenetic clock.^295^ More recently, a 13-day OSKM reprogramming using the Tet-on expression system significantly reduced the epigenetic age of human fibroblasts without fully changing them into iPSCs, indicating a boundary between the rejuvenation and the pluripotency programs.^296^ Furthermore, short-term expression of OSKM in vivo significantly expands the lifespan of progeria mice and restores the levels of H3K9me3 and H4K20me3.^292,297^ In addition, a 2.5-week transient reprogramming in early life (2-month-old mice) is sufficient to extend the lifespan of transgenic progeria mice by 15% and rejuvenates the DNA methylation patterns in skin cells.^298^ The aging-associated epigenetic and transcriptional changes can also be alleviated by transient reprogramming in naturally aged mice.^299^ Overall, both long-term and transient reprogramming can achieve the rejuvenation of aged cells, while transient reprogramming also provides a novel method to alleviate aging in vivo in an organism.

### Senolytic therapy

Senolytics selectively clear senescent cells in aged individuals and have been studied as a potential therapy for aging intervention. The first proposed senolytic strategy is the combination of dasatinib (D) and quercetin (Q), two pan-tyrosine kinase inhibitors.^29^ A single dose of D (5 mg/kg) + Q (50 mg/kg) effectively delays the aging phenotypes, such as frailty, cardiovascular diseases, and IVDD in aged mice, and extends the lifespan of *Ercc1*^*-/*△^ mice.^29^ To date, D + Q has been shown to prolong the healthspan and the physiological or pathological aging process in a variety of tissues or organs, including the cardiovascular system,^300,301^ skeleton,^302–304^ brain,^305,306^ adipose,^307,308^ lung,^309,310^ and muscle.^311^ Most recently, epigenetics regulation has been demonstrated to be an important mechanism by which D + Q eliminates aging cells. D + Q treatment leads to a significant change in epigenetic signatures in the hippocampus and improves the cognitive ability of aged male Wistar rats.^312^ Moreover, senescent adipose precursor cells exhibit hypomethylation and upregulated expression of the *ZMAT3* gene, which is related to type 2 diabetes; 3 days of D + Q treatment is able to increase DNA methylation of *ZMAT3* and decrease its expression, and reverse the senescence signature.^313^ In addition to D + Q, other senolytic drugs such as ABT-263, ABT-737, digoxin, FOXO4-DRI (D-retro inverso), and heat shock protein (HSP) 90 inhibitor 17-DMAG, play their senolytic roles mainly by inducing apoptosis and mitochondrial dysfunction,^314–318^ but their relationship to epigenetics needs further investigation.

### Active health intervention

**Fig. 9:**
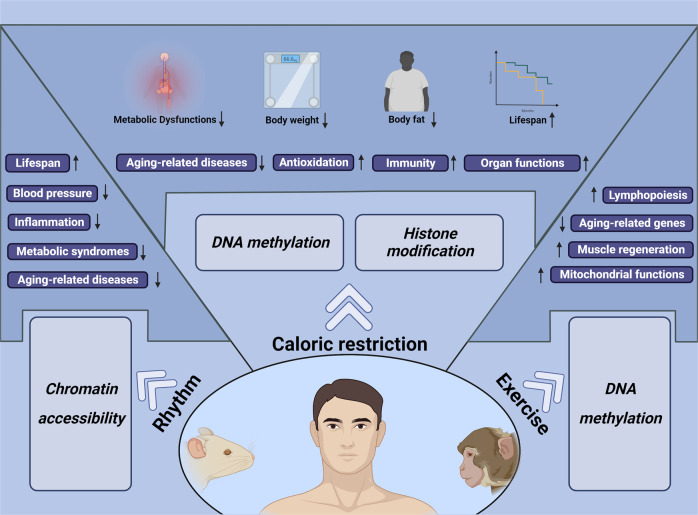
A healthy lifestyle to postpone aging. Active health, including caloric restriction, rhythm control and exercise, improves body function and affects the lifespan of various animals, suggesting that healthy lifestyles exert profound effects on aging intervention

Active health refers to choosing a healthy lifestyle autonomously, such as caloric restriction, regular routine and moderate exercise, which are considered to benefit the quality of life and may exert a rejuvenating effect on the aging process (Fig. 9). With the increasing awareness of active health, various studies have demonstrated that healthy lifestyles ameliorate aging-associated features in different animals and humans.

### Caloric restriction

CR, which reduces calorie intake ranging from 10 to 40%, has been demonstrated to expand the lifespan of rodents to varying degrees,^319–321^ attenuating vascular endothelial dysfunction, improving the aerobic function of skeletal muscles, and ameliorating the loss of muscle fibers and turnover of motor neurons.^322–325^ The effect of CR on lifespan and rejuvenation is at least partially due to the amelioration of aging-related epigenetic changes, such as DNA methylation and histone modification.^320,326^ It is likely that epitranscriptomic regulation also functions as an effector of CR. For example, CR in rats significantly inhibited the aging-associated down-regulation of the RNA m^6^A reader protein YBX1, which has been shown to be one of the drivers of stem cell aging.^327^ According to the epigenetic clock developed in mice, 40% CR treatment slows the molecular changes and reduces the epigenetic age in mouse livers. Notably, a 20-year CR, which reaches a final level at 30%, shows reduced aging-related pathologies and a significant lifespan expansion in adult rhesus monkeys, indicating that moderate CR also exerts a rejuvenating effect on primates.^328,329^ In humans, moderate CR slows biological aging, improves the function of the liver, and reduces oxidative stress and the incidence of aging-related diseases.^330–336^ In summary, CR has been widely proven to be an effective method to delay aging, and clinical trials demonstrate the accessibility to applying CR in humans, which will be discussed in the ‘Clinical intervention’ section later.

### Circadian rhythm

The circadian rhythm coordinates the behavior with the day/night shift and is also considered to play an essential role in the aging process.^337^ For example, epidermal and muscle stem cells from aged mice exhibit changed daily rhythms that cope with the stress of aging environments, indicating the continuous change of circadian rhythms along with the aging process.^338^ Disturbed circadian rhythm is linked to changes in chromatin structure, and it is found that 6 h sleep deprivation affects the chromatin accessibility in the cerebral cortex of mice, which contributes to long-term effects on gene expression.^339^ Forced circadian change may also accelerate the aging process and impair body function at a systemic level. Light schedule changes significantly affect aged mice, and advanced daytime leads to increased mortality of aged mice.^26^ In addition, mice with an innate circadian period close to 24 h live 20% longer than those with a shorter or longer innate circadian period.^340^ The disturbance of the circadian rhythm in rodents indicates that maintaining regular day/night cycles may reduce aging-related mortality and raise the question of whether circadian rhythm affects humans. Notably, a short-term circadian misalignment of 12-h inverted behavioral and environmental cycles for three days increases blood pressure and inflammatory markers in humans.^341^ However, how regular circadian rhythm benefits the healthspan, especially from the lens of epigenetic mechanisms, still needs further investigation.

### Exercise

Exercise may remodel DNA methylation on the promoter of key genes in skeletal muscle^342,343^ and histone modifications could also be changed by exercise through inhibition of the function of HDACs, thereby influencing the gene expression patterns.^344,345^ Moreover, exercise can modulate the expression of several miRNAs that mediate the beneficial process.^346,347^ After voluntary resistance training for 8 weeks, aged mice exhibit nearly 8 weeks of younger epigenetic age in their muscle and a modest lifespan extension.^56^ In addition, voluntary wheel running benefits aged mice in neurogenesis and learning ability^348^ and reduces the abnormal changes of the aged synapse.^324^ Importantly, the rejuvenating effect of exercise is also observed in humans. There is a significant difference in the transcriptional profile between physically active and sedentary aged adults, and endurance exercise improves the function of muscles in aged people.^349^ In addition, resistance training reduces the level of the mitochondrial methylome in aged human skeletal muscle and partially restores the aging-related change in the nuclear gene methylome in muscle.^350,351^ Clinical trials aiming to investigate the beneficial effects of exercise will be discussed in the “Clinical intervention” section later.

Current studies have demonstrated that a healthy lifestyle indeed exerts a beneficial effect on aging and therefore raises awareness of vibrant health. However, caloric restriction, circadian control and exercise all need to be moderate during implementation, and the boundary between healthy and unhealthy status requires further investigation. As our understanding of aging deepens, a healthy lifestyle is considered to be the easiest way for humans to interfere with aging, and active health definitively deserves more attention.

### Clinical intervention

Although various strategies targeting aging show satisfactory results in animal models, their effects in humans have yet to be demonstrated. Currently, diet intervention and exercise, which are extensively associated with epigenetic regulation, are the mostly studied and accepted strategies to target aging in humans. The clinical trial results demonstrate that CR (11.9%–25%) attenuates aging-related biomarkers, such as decreasing weight, enhancing insulin sensitivity and glucose tolerance, and improving major cardiometabolic risk factors.^334,352,353^ Time-restricted eating (TRE) in humans also provides benefits to some extent. Under an 8–10 h daily eating window of TRE, reductions in weight, blood pressure, atherogenic lipids, and cardiovascular risks are observed.^354,355^ A more stringent TRE (6 h window) also shows improvement in insulin sensitivity.^356^ However, the strategy of TRE is challenging to undertake, especially for cases with longer fasting times. Moreover, skipping breakfast has been found to be associated with an increased risk of mortality from cardiovascular disease.^357^ Therefore, an 11–12 h daily eating period is suggested to be ideal to avoid the compliance issues and side effects of TRE.^358^ Considering the difficulty for most subjects to adhere to chronic and extreme diets of CR or TRE, a fasting-mimicking diet (FMD) with low calories, low sugars, and low proteins but high unsaturated fats, provides another choice for a diet intervention. In a randomized phase 2 trial, healthy participants who received 3 monthly 5-day FMD cycles exhibited reduced markers/risk factors for aging, diabetes, cancer, and cardiovascular disease.^359^ In addition, a comprehensive understanding of the dietary interventions in humans has led to the proposal of the everyday normocaloric longevity diet that includes a mid to high carbohydrate and low but sufficient protein intake that is mostly plant-based but includes regular consumption of pesco-vegetarian-derived proteins.^358^ Diet intervention has also been found to be associated with epigenetic regulation in clinical trials. For example, CR in healthy and slightly overweight subjects significantly increases plasma concentrations of SIRT1.^360^ Five days of periodic fasting significantly elevated the expression of SIRT1 and SIRT3 in blood cells.^361^ Moreover, diet intervention has been shown to slow down the DNA methylation-based biomarkers of aging in several studies.^362,363^

Exercise has been demonstrated to be an effective geroprotector to improve the lifespan and healthspan in humans. Vigorous exercise, such as running, at middle and older ages, is associated with reduced disability in later life and reduced mortality,^364,365^ and leisure time physical activity of moderate to vigorous intensity is associated with longer life expectancy.^366^ In clinical intervention studies, exercise is found to reverse a series of aging-related diseases, including heart failure,^367–371^ cognitive decline,^372,373^ atherosclerosis,^374^ and insulin resistance.^375,376^ The geroprotective effect of exercise in humans is closely linked to epigenetic regulation. Exercise modifies the DNA methylation patterns in aged human skeletal muscle and reduces stochastic epigenetic mutations in crucial cancer-related pathways.^32,363^ Endurance exercise upregulates the expression of SIRT3 in the skeletal muscle and upregulates SIRT1, SIRT3, and SIRT6 in the serum.^375,377,378^ Exercise also modulates the microRNA expression profile (such as miR-423-3p, miR-451a, miR-766-3p, miR-130a, and miRNA-223) in subjects with type 2 diabetes, which may be involved in the improvement of weight loss, blood glucose control, and insulin sensitivity.^379–381^ In addition, the combination of diet and lifestyle interventions, including exercise, sleep, relaxation guidance, supplemental probiotics and phytonutrients, reverses the epigenetic age in healthy adult males.^362^

Pharmacological intervention is another major strategy to target natural aging. Epigenetic-related compounds, such as NMN, NR, and STACs, show potential as geroprotectors in clinical trials. Supplementation with NMN increases muscle insulin sensitivity, insulin signaling, and muscle remodeling in prediabetic women.^382^ NMN prevents aging-related muscle dysfunctions^383^ and shows benefits in improving aerobic capacity, cardiovascular fitness, sleep quality, fatigue, and physical performance.^89,384^ As for NR, clinical trials indicate that NR suppresses inflammatory activation of PBMCs in heart failure patients^385^ and decreases the levels of inflammatory cytokines in the serum and cerebrospinal fluid of Parkinson’s disease (PD) patients.^386^ However, most of these studies focus on the safety and tolerability of NR in patients.^386,387^ The effectiveness of NR in preventing or attenuating the progression of aging-related disorders should be verified in further studies, considering that several clinical trials show that NR does not improve insulin resistance.^388–391^ STACs exhibit inspiring effects in preclinical studies, but the results in clinical trials are not as satisfactory. For example, the natural STAC resveratrol and early synthetic STACs such as SRT1720 have very low bioavailability, potency, and limited target specificity,^392^ and other STACs, such as SRT2379 and SRT3025, produce no significant clinical responses. Similarly, although SRT2104 shows some benefits on lipid parameters, including cholesterol and triglycerides,^393,394^ it does not improve glucose or insulin control^394,395^ and has no significant anti-inflammatory effect in ulcerative colitis patients.^396^ The poor and variable pharmacokinetics upon oral administration of SRT2104 need to be resolved in the future.

Other geroprotective interventions, such as metformin, rapamycin, and D + Q, have also been explored in clinical trials, and the data show that metformin administration reduces the incidence of diabetes,^397^ cardiovascular events,^398^ frailty,^399^ and cognitive impairment,^400^ and improves putative longevity effectors in PBMCs.^401^ Although rapamycin shows exciting effects in preclinical studies, similar results have not been observed in clinical trials. In addition, despite the improved immune function in the elderly after administration of the mTOR inhibitor RAD001,^402,403^ several studies show that rapamycin does not improve cognitive function or physical performance^404^ and does not improve frailty.^405^ The first clinical study of senolytics demonstrated that D + Q improves 6-min walk distance, walking speed, chair raise ability, and short physical performance battery in idiopathic pulmonary fibrosis (IPF) patients.^309^ D + Q also reduces senescent cell burden in adipose tissue and skin and reduces circulating SASP in people with diabetic kidney disease.^406^

Collectively, diet intervention and exercise are still the most accepted strategies to intervene in aging and aging-related diseases, mainly because of their effectiveness and safety for humans. Advances in pharmacological interventions such as geroprotectors also show improvement in multiple aging-related conditions; however, the safety concerns and inconsistent results of these strategies demand further clinical evidence. Many other clinical trials related to epigenetic targets and the regulations of aging are still ongoing (Table 1). Together, these findings will provide more candidates for gerotherapeutics and pave the way for fighting aging in the future.

**Table 1:** Ongoing clinical trials related to epigenetic targets and regulation of aging

Interventions	Conditions/diseases	Trial nO.	Phase
Diet intervention	Epigenetic aging	NCT04962464	2
	Epigenetic aging	NCT05297097	2
	Epigenetic aging	NCT05234203	NA
	Aging	NCT05424042	NA
Exercise	Aging	NCT05232968	NA
	Aging; Alzheimer disease	NCT04299308	NA
	Biological aging	NCT03440099	NA
	Aging; Inflammatory response	NCT05042167	NA
NMN	Glucose metabolism disorders	NCT04571008	NA
	Hypertension	NCT04903210	4
	Physical activity; Muscle recovery	NCT04664361	NA
NR	Aging	NCT03818802	NA
	Parkinson disease	NCT03568968	NA
	Overweight and obesity; Aging; Type 2 diabetes	NCT04907110	NA
	Sarcopenia; Nicotinamide adenine dinucleotide concentration; Muscle quality and NAD^+^ content	NCT04691986	NA
STACs	Vascular resistance; Hypertension	NCT01842399	1/2
	Healthy	NCT00996229	3
Metformin	Epigenetic aging; Immunosenescence	NCT04375657	2
	Aging; Insulin sensitivity; Chronic disease; Mitochondria; Insulin resistance	NCT04264897	3
	Frailty; Sarcopenic obesity; Aging	NCT04221750	3
	Frailty	NCT02570672	2
	Mild cognitive impairment	NCT04098666	2/3
	Insulin resistance; Obesity	NCT03733132	2
Rapamycin	Epigenetic clock of skin	NCT04608448	1
	Aging	NCT04488601	2
	Aging	NCT04742777	2
	Mild cognitive impairment; Alzheimer disease	NCT04629495	2
	Mild cognitive impairment; Alzheimer disease	NCT04200911	1
D + Q	Epigenetic aging	NCT04946383	2
	Diabetic kidney disease	NCT02848131	1
	Alzheimer disease	NCT04063124	1/2
	Mild cognitive impairment; Alzheimer disease	NCT04785300	1/2
	Alzheimer disease; Mild cognitive impairment	NCT04685590	2
	Aging-related osteoporosis	NCT04313634	2

## Conclusion and perspective

Studies in *C. elegans*, *Drosophila*, and mammals have unraveled the aging-related epigenetic changes in DNA, RNA, and histone modifications and alterations in the more advanced chromatin structure states. Correspondingly, these epigenetic changes have been identified as biomarkers or intervention targets of aging, such as the global decrease in genomic DNA methylation, the global loss of canonical histones, chromatin landscape remodeling caused by heterochromatin loss, and nuclear membrane protein changes in human and mouse tissues during aging. However, the same chromatin modifications (e.g., H3K14ac and H3K27me3) may play opposite roles in regulating aging and longevity across species and even across tissues within the same species, indicating that epigenetic changes need to be interpreted with their context. It is noteworthy that emerging technologies such as single-cell omics sequencing provide a higher resolution for dissecting epigenetic characteristics during aging, and provide new avenues for investigating the heterogeneity of aged cells. In addition, the spatiotemporal transcriptomic atlas across multiple mammalian tissues can provide more information on aging-related interactions between cells or tissues, which may facilitate the design of better and more precise therapeutics for aging and aging-related diseases.

Based on these epigenetic changes in cells during aging, a series of corresponding therapeutic strategies have been developed. Geroprotective drugs targeting longevity-related histone acetylation, including supplementation with NAD^+^ precursors, STACs have been tested in various species. Metformin, rapamycin, and other drugs have also shown positive effects in alleviating aging-related pathologies and regulating aging-related epigenetic changes in preclinical studies; however, the safety and efficacy of these drugs require more clinical investigation. Currently, a rational diet and exercise are considered the most effective and easiest way to delay aging, but drugs targeting key aging-related molecular and cellular changes are still promising and attractive clinical treatment strategies for intervening in aging and treating aging-related diseases.

Despite all the recent progress, it remains unclear how epigenetic changes interact with other factors, including the genetic background and even the microbiome, to regulate the aging process. It is also unclear how environmental factors, lifestyles, and physiological and psychological states contribute to epigenetic changes in the aging process. Furthermore, as aging is a continuous process that occurs over many years in humans, it would be necessary to track the epigenetic changes in this entire process for a better understanding of what and how epigenetic regulations contribute to each stage of aging.
